# Psychometric properties of the nine-item avoidant/restrictive food intake disorder screen (NIAS) in Turkish children

**DOI:** 10.1186/s40337-024-00987-0

**Published:** 2024-02-19

**Authors:** Hakan Öğütlü, Meryem Kaşak, Uğur Doğan, Hana F. Zickgraf, Mehmet Hakan Türkçapar

**Affiliations:** 1 Department of Child and Adolescent Psychiatry, Cognitive Behavioral Psychotherapies Association, Karum Is Merkezi Iran Caddesi No: 21 Gaziosmanpasa Mah., 06680 Cankaya, Ankara, Turkey; 2 Department of Child and Adolescent Psychiatry, Ankara City Hospital, Ankara, Turkey; 3 Departman of Guidance and Counseling, Muğla Sıtkı Koçman University, Muğla, Turkey; 4 Department of Psychology, University of South Alabama, Mobile, AL USA; 5 Department of Pediatrics, Emory University, Atlanta, GA USA; 6 Rogers Behavioral Health, Oconomowoc, WI USA; 7 Department of Psychology, Social Sciences University of Ankara, Ankara, Turkey

**Keywords:** NIAS, ARFID, Avoidant/restrictive food intake disorder, Validation, Children

## Abstract

**Background:**

The nine item avoidant/restrictive food intake disorder screen (NIAS) is a short and practical assessment tool specific to ARFID with three ARFID phenotypes such as “Picky eating,” “Fear,” and “Appetite”. This study aimed to evaluate the psychometric properties of the Turkish translation of the NIAS parent form and to investigate the relationship between ARFID symptoms and anxiety, depression symptoms, and eating behaviors in a sample of Turkish children.

**Method:**

Parents were asked to provide their children's sociodemographic data and to complete the NIAS, Eating Disorder Examination Questionnaire-Short (EDE-QS), Children's Eating Behavior Questionnaire (CEBQ), and Revised Child Anxiety and Depression Scale (RCADS) scales.

**Results:**

The sample included 440 participants between 6 and 12 ages. Turkish NIAS demonstrated good internal consistency. The three-factor model of the Turkish NIAS was in an acceptable structure. The Turkish NIAS scale was shown to be valid and reliable. NIAS scores were shown to be higher in underweight participants. The NIAS-parent version subscales showed expected convergent and divergent validity with the CEBQ, EDEQ-S, and RCADS scales in children, except CEBQ emotional overeating and desire to drink subscales were correlated with NIAS.

**Conclusion:**

The Turkish version of the NIAS is valid and reliable in evaluating ARFID symptoms in children.

## Abstract (plain language summary)

Assessment tools for avoidant/restrictive food intake disorder (ARFID), which is quite common in children, are quite limited. NIAS-parent version is a practical and valuable scale that can be used in the clinic. This study found that the Turkish version of the NIAS is valid and reliable in evaluating ARFID symptoms in children. In the psychometric properties of the Turkish NIAS, ARFID symptoms were associated with anxiety and depression symptoms and food-avoidant eating behavior. It was also found that ARFID was not associated with eating disorder symptoms. In addition, the paper shows initial data concerning the psychometric properties related to the Turkish NIAS-parent version. It is the first study to evaluate the relationship of ARFID subtypes with anxiety, depression symptoms, appetite characteristics, and BMI percentages in children.

## Background

Avoidant/restrictive food intake disorder (ARFID) is characterized by food avoidance or dietary restriction that causes significant weight loss, growth retardation in childhood, dependence on nutritional supplements (i.e., oral or enteral formulas), or impairment in psychosocial functioning [1]. Although ARFID was not a new condition and has been observed by clinicians for a long time, it was first introduced to psychiatric nosology with the publication of DSM-5 in 2013 [1]. It is worth noting that prior to being labeled as ARFID, clinicians referred to similar conditions using different terms, including but not limited to infantile anorexia and feeding disorder of infancy and early childhood [2].

Thomas et al. [3] proposed a three-dimensional model of neurobiological abnormalities in sensory perception, homeostatic appetite regulation, and negative valence systems that underlie the three essential aspects of ARFID: selective/neophobic eating, lack of interest in foods and eating, and fear of aversive consequences, respectively. Parental inability to model appropriately (for example, no family meals, skipping meals, watching TV during meals, parents’ disordered eating symptoms), parents' authoritarian or permissive feeding practices, and limited exposure to food may play a role as environmental risk factors [4, 5]. However, parental behaviors have not been extensively studied in ARFID, and limited longitudinal data are available to assess the direction of the relationship between familial factors and child ARFID symptoms.

It is essential to detect ARFID, which causes significant psychosocial and medical problems, in the clinic, but screening tools are minimal and not specific to diagnosing ARFID. Cooney et al. [6] stated that psychometric measurement tools widely used to evaluate eating disorders are not sensitive and specific for diagnosing ARFID in the pediatric group.

The NIAS is a short and practical assessment tool measuring the degree to which each ARFID phenotype, such as “Picky eating," "Fear," and "Appetite" is experienced [7]. A parent-report version of the NIAS for children ages five and up has been developed and is used clinically. Still, to our knowledge, only the selective eating subscale has been used in published empirical research [8, 9]. The NIAS scale has been translated into multiple languages [10] after being validated in a large population sample in the United States [7], but no Turkish validity study was performed. In addition, as far as is known, no study comprehensively evaluated the relationship of all three ARFID phenotypes with anxiety, depression symptoms, and eating behaviors in children using the NIAS.

Therefore, this study aimed to evaluate the psychometric properties (factor structure, reliability, and convergent and divergent validity) of the Turkish version of the NIAS parent form, to determine the validity of the scale in Turkish children, and to investigate the relationship between ARFID symptoms and body weight (body mass index percentile), weight/shape-related disordered eating, anxiety, and depression symptoms and appetitive traits linked to body weight in childhood. Based on patterns observed using the adult self-report NIAS (e.g., [7]) we predicted that the picky eating and appetite subscales would be negatively related to appetitive traits associated with food approach, including food responsiveness and enjoyment of eating. Based on findings that ARFID is more commonly comorbid with anxiety than depression, we predicted that all subscales would be related to overall anxiety. Finally, we predicted that the NIAS subscales would be uncorrelated with a measure of weight/shape-related disordered eating symptoms and that the appetite subscale would be negatively correlated with the BMI percentile [7].

## Methodology

### Participants

A population sample of children aged 6–12 was used in the study. There were no exclusion criteria in the study. The study sample was taken from primary and secondary schools (i.e., 1st to 7th grades) in Muğla, Turkey. In order to prevent selection bias, the school and class selections of the participants in the study were provided by the Muğla Guidance and Research Center using the blind method in selecting the study group; the non-random, convenient sampling method was adopted. Of the 734 participants in the selected classes, 266 (36.2%) parents did not consent to the study, and 28 (3.8%) participated but did not complete all of the scale items. The final sample included 440 participants, 217 (49.31%) girls and 223 boys (50.68%), with a mean age of 9.08 ± 1.9 years. Mothers (n = 382, 86.8%), fathers (n = 44, 10%), and other relatives (n = 14, 3.18%) completed the ratings. The mean weight of the children was 32.74 kg (± 10.55), height was 134.45 cm (± 14.04), Body Mass Index (BMI) was 17.73 (± 3.35), and percentiles were 58.1 (± 33.22).

Upon evaluating the cut-off scores of children using the Revised Child Anxiety and Depression Scale (RCADS), it was found that 92.0% of the sample (405 children) were clinically normal for depression, while 8% (35 children) were at the clinical level. In terms of anxiety, 80.9% (356 children) were classified as normal, and 19.1% (84 children) were at the clinical level. The breakdown of clinical levels for different anxiety subtypes is as follows: Generalized Anxiety—16.4% (72 children) clinical, Panic—18.9% (83 children) clinical, Separation Anxiety—23.9% (105 children) clinical, Social Phobia—14.5% (64 children) clinical, and Obsessions/Compulsions—18.9% (83 children) clinical, with 76.1% (335 children) being normal.

For test–retest reliability, 70 randomly selected participants were asked to re-answer the NIAS one month after the initial administration. Fifty-two participants (74.2%) answered the items again.

### Procedure

Permission was obtained from the developer of NIAS to adapt the scale to Turkish children via e-mail. Ethics committee approval was obtained for the study from the Social Sciences University of Ankara Institutional Ethics Committee of Social Sciences and Humanities Research and Publication (No:2022/44352, Date: 08/08/2022). Then, in the first stage, the English version of the NIAS was translated into Turkish by two Turkish clinical psychologists with a good command of English. These two psychologists translated the scales independently, followed by consensus after a discussion between the two translators and the corresponding authors of this article. Next, a professional bilingual translator who had no previous knowledge of the scale translated the Turkish form of the scale back into English. The NIAS developer later reviewed the translated version, and some minor wording changes were made. Afterward, the final version of the scale was applied to the parents of 10 children, the researchers corrected misunderstandings, and the Turkish version was given its final form.

Next, an online survey was created using the Survey Monkey website (www.surveymonkey.com) to be administered to the participants. On the first page of the questionnaire, the content and objectives of the study were detailed, and all the necessary information for informed consent was given to the parents. Informed consent was obtained from the participants. In the questionnaire, parents were asked to provide their children's socio-demographic (i.e., age, gender), weight, and height data and to complete the NIAS, Eating Disorder Examination- Questionnaire-parent (EDE-QS-parent), Children's Eating Behavior Questionnaire (CEBQ), and RCADS scales.

### Measurements

#### Nine item avoidant/restrictive food intake disorder screen (NIAS)

Developed by Zickgraf & Ellis (2018) [7], the NIAS is a 9-item self-report scale that evaluates avoidant/restrictive eating disorder symptoms. The NIAS has three subscales: Picky eating (items 1–3), Appetite (items 4–6), and Fear (items 7–9). Each item of the NIAS is scored between 0 (‘strongly disagree’) and 5 (‘strongly agree’). Each subscale is scored on a scale of 0–15, with higher scores indicating higher levels of each subscale. All items can also be added together to calculate an overall score ranging from 0 to 45, with higher scores indicating higher levels of avoidant/restrictive eating overall. In the original study, Cronbach's α value for the NIAS total score to assess internal reliability was 0.90 [7]. In this study, Cronbach’s α was 0.81 for the NIAS total score.

#### Eating disorder examination-questionnaire short (EDE-QS)

The EDE-QS is a 12-item single-factor scale developed by Gideon et al. [11] that evaluates the core symptoms of AN, BN, and eating disorder not otherwise specified (EDNOS). It is the abbreviated form of EDE-Q [12]. Each item of the EDE-QS is scored between 0 (0 days/Not at all) to 3 (6–7 days/Markedly), and a total score is obtained by summing and averaging the items; higher scores indicate more severe levels of eating disorders. In the original study, Cronbach's α value for the EDE-QS total score was 0.91. The validity and reliability study of the Turkish version of this scale in an adult sample was conducted by Esin et al. [13]. In this study, Cronbach's α value for the Turkish version of the parent version of EDE-QS total score was 0.88.

#### Children’s eating behavior questionnaire (CEBQ)

This scale was developed by Wardle et al. [14] to determine children's eating behaviors. The scale, answered by the parents, consists of 35 items. Each item of the CEBQ is scored between 1 (‘never’) and 5 (‘always’). The CEBQ has eight subscales: food responsiveness (FR), emotional overeating (EOE), enjoyment of food (EF), desire for drinks (DD), satiety responsiveness (SR), slowness in eating (SE), emotional undereating (EUE), and food fussiness (FF). Yılmaz et al. [15] carried out the Turkish validity of the scale. The Cronbach’s α values for the CEBQ ranged from 0.70 to 0.89 in the original study [14].

#### Revised child anxiety and depression scale (RCADS)

RCADS was developed by Chorpita et al. [16] to screen for anxiety disorders and depression in children and adolescents. The scale, which has two versions as child and parent forms, consists of 47 items. Each item of the RCADS is scored between 0 (‘never’) and 3 (‘always’). The RCADS has six subscales: generalized anxiety disorder (GAD) (6 items), separation anxiety disorder (SPAD) (9 items), social anxiety disorder) (SAD) (7 items), panic disorder (PD) (9 items), obsessive–compulsive disorder (OCD) (6 items), and major depressive disorder (MDD) (10 items). Görmez et al. [17] carried out the Turkish validity of the scale. The Cronbach’s α values for the RCADS ranged from 0.78 for SAD to 0.88 for GAD in the original study [16].

#### Body mass index (BMI)

BMI was calculated (kg/m2), and the BMI percentiles for age were determined using World Health Organisation (WHO) growth charts. Based on the recommended cutoff points of BMI percentiles for Turkish children and adolescents [18] (i.e., < 5 percentiles = underweight, 5–85 percentiles = normal weight, > 85 percentiles = overweight). Parents of the participants reported their children’s weight and height, which were used to compute BMI. The BMI values of the participants were used in the study because they were related to the A.1 criterion (weight loss due to restrictive eating) of ARFID diagnosis according to DSM-5.

### Data analysis

Statistical analyses of scale adaptation were made with the JASP (2020) [19] program. JASP is software built on the R (R Core Team, 2019) [20] program, using R packages [21]. The student’s t-test and ANOVA were performed for the genders and BMI percentile categories of the subscale scores and total scores of the NIAS. In cases where significant differences were detected, post hoc analysis (Bonferroni) was performed to determine the source of the difference between the groups.

Confirmatory factor analysis (CFA) was used in the data analysis to test the construct validity. For the CFA analysis at scale, the JASP program uses the “lavaan” [22], “semPlot” [23], and “psych” [21] packages based on the CFA analysis assumptions in Brown (2014) [24] and Kline's (2015) (35) books. For CFA, one of the estimation methods, “Maximum likelihood” was used. The studied data meet the three basic assumptions of Maximum likelihood: (1) the sample is large, (2) the data is continuous, and (3) it requires multivariate normally distributed indicators. Fit indices were evaluated as a result of CFA, according to recommended values for an adequate model fit based on the literature: Chi-squared statistic/degrees of freedom (χ2/df) < 5, root-mean-square error of approximation (RMSEA) < 0.08; Goodness-of-Fit Index (GFI) > 0.90 [25], Comparative Fit Index (CFI) ≥ 0.95, Standardized Root Mean Square Residual (SRMR) ≤ 0.08 [26, 27], Tucker–Lewis index (TLI) ≥ 0.90 [26].

Pearson correlation test was used to examine the relationship between “Picky eating” (NIAS-picky eating), “Fear” (NIAS-fear), and “Appetite” (NIAS-appetite) subscale scores of NIAS, EDE-QS, CEBQ, and RCADS scores, and children's BMIs. In addition, partial correlation analysis was conducted to control variables.

Item-total correlation, test–retest, and Cronbach’s α internal consistency coefficient were used for the reliability analysis. Item-total correlation and test–retest (initial and follow-up scores of NIAS) were performed with Pearson’s correlation test. The mean values of items were expressed with standard deviation, and results with *p* < 0.05 are considered statistically significant.

## Results

### Factor analysis of the NIAS

The averages of the items of the NIAS ranged from 0.61 to 2.89. The means and standard deviations of the items on the scale are shown in Table 1.

**Table 1:** Descriptive statistics, adjusted ıtem-total correlation and Cronbach’s α value of NIAS

Dimension	Items	Mean	SD	Item Total Correlation	IIDCα	t-test	Cα
Picky eating	NIAS 1	2.89	1.54	0.55	0.79	− 17.49*	0.84
	NIAS 2	2.00	1.53	0.59	0.78	− 19.42*	
	NIAS 3	1.85	1.62	0.60	0.78	− 20.35*	
Appetite	NIAS 4	1.63	1.53	0.63	0.78	− 18.73*	0.81
	NIAS 5	2.11	1.60	0.57	0.79	− 20.01*	
	NIAS 6	1.28	1.37	0.45	0.80	− 13.26*	
Fear	NIAS 7	0.61	1.02	0.40	0.81	− 8.22*	0.87
	NIAS 8	0.70	1.07	0.41	0.81	− 8.53*	
	NIAS 9	0.63	0.99	0.37	0.81	− 8.40*	
Full scale							0.81

*NIAS* Nine Item Avoidant/Restrictive Food Intake Disorder Screen, *SD* Standard Deviation, *IIDCα* If Item Dropped Cronbach’s α; *t-test* t-test for Distinguishing Features of Items, *Cα* Cronbach’s α

t Test*p < 0.05

### Construct validity

#### Confirmatory factor analysis (CFA)

First-level CFA was performed to determine the construct validity to adapt the NIAS scale to the Turkish language. It was seen that the three-dimensional model of the measurement tool was confirmed in the CFA result of the NIAS scale. When the fit indices related to the analysis were examined, it was seen that it gave excellent results (χ2 = 59.3, df = 24, χ2/df = 2.47; RMSEA = 0.058 [Cl lower = 0.039, Cl upper = 0.076]; CFI = 0,981; TLI = 0,971; SRMR = 0.037). The standardized factor loading values were 0.74 and 0.91 on the whole scale. It was seen that all factor loads are significant, and the residual covariances of the items are not high. Factor loadings and the CFA results of the scale are given in Table 2.

**Table 2:** Factor loadings and CFA results of NIAS

	Items	Estimate	SE	%95 CI		St. Est	RC
				Lower	Upper		
Picky eating	NIAS 1	1.14*	0.06	1.00	1.27	0.74	0.45
	NIAS 2	1.28*	0.06	1.15	1.40	0.84	0.30
	NIAS 3	1.35*	0.06	1.22	1.49	0.84	0.30
Appetite	NIAS 4	1.24*	0.06	1.11	1.37	0.81	0.34
	NIAS 5	1.36*	0.07	1.22	1.49	0.85	0.28
	NIAS 6	0.87*	0.06	0.75	0.10	0.64	0.59
Fear	NIAS 7	0.81*	0.04	0.73	0.89	0.80	0.36
	NIAS 8	0.85*	0.04	0.76	0.94	0.79	0.37
	NIAS 9	0.90*	0.04	0.82	0.98	0.91	0.17

*NIAS* Nine Item Avoidant/Restrictive Food Intake Disorder Screen, *SE* Standard Eror, *CI* Confidence Interval; *RC* Residual Covariances, *St.Est*: Standart Estimate

*p < 0.05

For the CFA, the covariances between the factors were calculated, and the results are given in Table 3. It was seen that the covariance between NIAS-picky eating and NIAS-appetite is 0.55, the covariance between NIAS-fear and NIAS-picky eating is 0.20, and the covariance between NIAS-appetite and NIAS-fear is 0.24.

**Table 3:** Factor covariance of NIAS

	Est	SE	95% CI	
			Lower	Upper
Picky eating				
Picky eating	1			
Appetite	0.55***	0.04	0.46	0.63
Fear	0.20***	0.05	0.10	0.31
Appetite				
Appetite	1			
Fear	0.24***	0.05	0.14	0.34
Fear				
Fear	1			

*NIAS* Nine Item Avoidant/Restrictive Food Intake Disorder Screen, *SE* Standard Eror, *CI* Confidence Interval

^***^p < 0.001

### Descriptive analysis of the NIAS

First, the dimensions and total scores of NIAS were defined and compared according to the BMI percentile category and gender. Specifically, for BMI, underweight participants showed significantly higher scores on the NIAS full-scale score and NIAS-appetite than those in the healthy weight and overweight/obese range (all *p* < 0.05). There were no significant gender differences in any subscale or the total, score. The results are shown in Table 4.

**Table 4:** Descriptive Analysis and Group Comparisons of NIAS by BMI and Gender

	Underweight (n = 97) M (SD)	Normal (n = 193) M (SD)	Overweight (n = 150) M (SD)	F	Post-Hoc
Picky eating	7.10 (± 4.18)	6.77 (± 3.67)	6.53 (± 4.36)	0.63	–
Appetite	6.22 (± 4.04)	5.42 (± 4.18)	4.10 (± 3.53)	11.58*	UW > N > OW
Fear	2.03 (± 3.06)	1.96 (± 2.65)	1.92 (± 2.74)	0.05	–
NIAS total	15.35 (± 8.80)	14.15 (± 7.69)	12.55 (± 7.45)	4.45*	UW > N > OW

	Girls (n = 217) M (SD)	Boys (n = 223) M (SD)	t
Picky eating	6.76 (± 4.09)	6.72 (± 4.11)	0.10
Appetite	5.38 (± 3.93)	4.67 (± 3.71)	1.95
Fear	1.94 (± 2.79)	1.97 (± 2.78)	− 0.12
NIAS total	14.08 (± 7.91)	13.36 (± 7.90)	0.95

*NIAS* Nine Item Avoidant/Restrictive Food Intake Disorder Screen, *BMI* Body Mass Index, *SD* Standard Deviation, *UW* Underweight, *N* Normal, *OW* Overweight

^*^p < 0.05

### Criterion validity

The criterion validity of the NIAS was assessed according to the correlation between subscale scores and BMI percentile values, CEBQ, EDE-QS, and RCADS scores. There was no significant relationship between NIAS total score and EDE-QS (r = 0.06, *p* = 0.22). There was a negative and significant relationship between the NIAS total score and BMI (r = − 0.17, *p* < 0.001) of the children. Finally, there were positive and significant relationships between the NIAS total score and the RCADS all anxiety sub-dimensions scores, the total anxiety score (r = 0.23, *p* < 0.001), and the depression score (r = 0.34, *p* < 0.001).

Table 5 shows the correlation results of the three NIAS subscales with each variable. While a small-moderate and negative relationship existed between BMI percentile and NIAS-appetite, there was no significant relationship between other subscales and BMI. NIAS-appetite and NIAS-fear showed a small and significant correlation with parent-reported weight/shape eating disorder symptoms on the EDEQ-S. In contrast, NIAS-picky eating had no relationship with non-ARFID eating disorder symptoms. Overall, NIAS-appetite showed the expected pattern of relationships with the CEBQ subscales, with positive and strong relationships with SR and SE and moderate-large negative relationships with EF and FR. The relationships between NIAS-Appetite and EUE/EOE were small-moderate but significant and in the expected direction, and there was a small but significant positive relationship with FF. Also, as predicted, NIAS-picky eating was most strongly correlated with the CEBQ food fussiness scale. The relationships between NIAS-picky and the other CEBQ subscales mirrored those of NIAS-appeite, with generally smaller effect sizes. The exceptions were a null relationship with food responsiveness (FR) and a strong relationship with satiety responsiveness (SR). NIAS-fear had small but significant positive associations with EOE, DD, SR, SE, and EUE and a null relationship with FR and FF. All three subscales were positively associated with overall Depression and Anxiety symptoms on the RCADS; effect sizes for NIAS-picky eating and NIAS-appetite were small, whereas the relationships with NIAS-fear were moderate. Of the RCADS symptom subscales, NIAS-picky eating had the strongest relationship (r = 0.31) with OCD symptoms. In contrast, NIAS-fear had the strongest relationships with panic disorder (r = 0.33) and MDD (r = 0.33). All three subscales had small, positive relationships with SAD symptoms. Still, only Fear was significantly associated with GAD, with a small effect size.

**Table 5:** Convergent and divergent validity of the NIAS

Scales	NIAS	Pearson's r	*p*	Pearson's r(Controlling for EDE-QS)	*p*
EDE-QS	Picky_Eating	0.04	0.389	–	–
	Appetite	− 0.11*	0.028	–	–
	Fear	0.25***	< 0.001	–	–
BMI Percantil	Picky_Eating	− 0.07	0.160	− 0.08	0.105
	Appetite	− 0.26***	< 0.001	− 0.24***	< 0.001
	Fear	− 0.03	0.477	− 0.09	0.062
CEBQ					
Food responsiveness	Picky_Eating	− 0.05	0.316	− 0.07	0.169
	Appetite	− 0.38***	< 0.001	− 0.37***	< 0.001
	Fear	0.05	0.303	− 0.02	0.731
Emotional overeating	Picky_Eating	− 0.02	0.747	− 0.04	0.444
	Appetite	− 0.29***	< 0.001	− 0.27***	< 0.001
	Fear	0.12**	0.003	0.09	0.064
Enjoyment of food	Picky_Eating	− 0.37***	< 0.001	− 0.38***	< 0.001
	Appetite	− 0.65***	< 0.001	− 0.65***	< 0.001
	Fear	− 0.11*	0.032	− 0.14**	0.004
Desire to drink	Picky_Eating	0.11*	0.023	0.11*	0.031
	Appetite	− 0.01	0.825	0.01	0.943
	Fear	0.11*	0.025	0.08	0.092
Satiety responsiveness	Picky_Eating	0.53***	< 0.001	0.53***	< 0.001
	Appetite	0.53***	< 0.001	0.53***	< 0.001
	Fear	0.10**	0.004	0.17***	< 0.001
Slowness in eating	Picky_Eating	0.29***	< 0.001	0.29***	< 0.001
	Appetite	0.47***	< 0.001	0.47***	< 0.001
	Fear	0.12**	0.004	0.18***	< 0.001
Emotional undereating	Picky_Eating	0.19***	< 0.001	0.18***	< 0.001
	Appetite	0.17***	< 0.001	0.19***	< 0.001
	Fear	0.13**	0.007	0.10*	0.036
Food fussiness	Picky_Eating	0.48***	< 0.001	0.49***	< 0.001
	Appetite	0.19*	0.028	0.29***	< 0.001
	Fear	0.01	0.821	0.14**	0.004
RCADS					
SAD	Picky_Eating	0.17***	< 0.001	0.17**	0.001
	Appetite	0.18***	< 0.001	0.19***	< 0.001
	Fear	0.16**	0.003	0.13*	0.010
GAD	Picky_Eating	0.08	0.109	0.06	0.219
	Appetite	0.10	0.060	0.11	0.055
	Fear	0.22***	< 0.001	0.14**	0.009
MDD	Picky_Eating	0.22***	< 0.001	0.22***	< 0.001
	Appetite	0.23***	< 0.001	0.30***	< 0.001
	Fear	0.33***	< 0.001	0.25***	< 0.001
PD	Picky_Eating	0.04	0.489	0.01	0.851
	Appetite	0.14**	0.009	0.19***	< 0.001
	Fear	0.31***	< 0.001	0.23***	< 0.001
SPAD	Picky_Eating	0.11*	0.036	0.09	0.079
	Appetite	0.09	0.085	0.13*	0.010
	Fear	0.23***	< 0.001	0.15**	0.005
OCD	Picky_Eating	0.31***	< 0.001	0.25***	< 0.001
	Appetite	0.10	0.066	0.14*	0.055
	Fear	0.07	0.178	0.05	0.338
Depression	Picky_Eating	0.22***	< 0.001	0.22***	< 0.001
	Appetite	0.23***	< 0.001	0.30***	< 0.001
	Fear	0.33***	< 0.001	0.25***	< 0.001
Anxiety Total	Picky_Eating	0.12*	0.021	0.10*	0.048
	Appetite	0.15**	0.005	0.20***	< 0.001
	Fear	0.29***	< 0.001	0.21***	< 0.001
Total	Picky_Eating	0.15**	0.004	0.14**	0.009
	Appetite	0.17***	< 0.001	0.23***	< 0.001
	Fear	0.31***	< 0.001	0.23***	< 0.001

*NIAS* Nine Item Avoidant/Restrictive Food Intake Disorder Screen, *EDE-QS* Eating Disorder Examination- Questionnaire Short, *BMI* Body Mass Index, *CEBQ* Children's Eating Behaviour Questionnaire, *RCADS* Revised Child Anxiety and Depression Scale, *SAD* Social Anxiety Disorder, *GAD* Generalized Anxiety Disorder, *MDD* Major Depressive Disorder, *PD* Panic Disorder, *SPAD* Seperation Anxiety Disorder, *OCD* Obsessive Compulsive Disorder, *Total* Total Anxiety&Depression

^*^p < 0.05,   ^**^p < 0.01, ^***^p < 0.001

Partial correlation analysis was performed to control the EDEQ-S score for the relationship between NIAS scores and BMI percentile, CEBQ subscales, and RCADS scores. Again, while a negative and significant relationship was found between BMI percent and NIAS-appetite (r = − 0.27, *p* < 0.001), no significant relationship was found between other subscales and BMI (*p* > 0.05). When symptoms of eating disorders other than ARFID were controlled, the negative and significant relationship between NIAS-appetite and CEBQ's food approach subscales (FR, EOE, and EF) and the positive and significant relationship with food avoidance subscales (SR, SE, and EUE) continued. NIAS-picky eating was also positively and strongly associated with FF (r = 0.49, *p* < 0.001). Again, all three subscales remained positively associated with overall Depression and Anxiety symptoms on the RCADS (*p* < 0.001) (Table 5).

### Reliability analysis of the NIAS

#### Internal consistency

The internal consistency of the scale was tested by using Cronbach’s α value. Cronbach’s α was 0.81 for the NIAS total score, 0.84 for Picky Eating, 0.81 for Appetite, and 0.87 for Fear. It can be said that the values between 0.80 and 0.90 that emerged as a result of the analyses have “good reliability” [28].

#### Distinguishing features of items

Another way to ensure reliability is to compare the lower 27% and upper 27% groups. Since the lower 27% and upper 27% groups formed according to the total scores obtained from the measurement tool are expected to be different in terms of the measured feature, there is expected to be a significant difference between the item average scores of the groups. The t-test was conducted to determine the significance of the differences between the item average scores of the upper 27% (N:118) and lower 27% (N:118) groups from the study population. According to the t-test results of the groups, it was found that there was a significant (*p* < 0.001) difference between the lower and upper groups. Regarding this result, the items have good discrimination. The t values for the analysis are given in Table 1.

#### Item analysis

The item-total correlation results of the scale ranged from 0.55 to 0.60 for Picky eating, 0.45 to 0.63 for Appetite, and 0.37 to 0.41 for Fear. The fact that the items had high correlations with the scale scores and each other indicates a potential hierarchical latent structure. Values related to the analysis are given in Table 1.

**Fig. 1:**
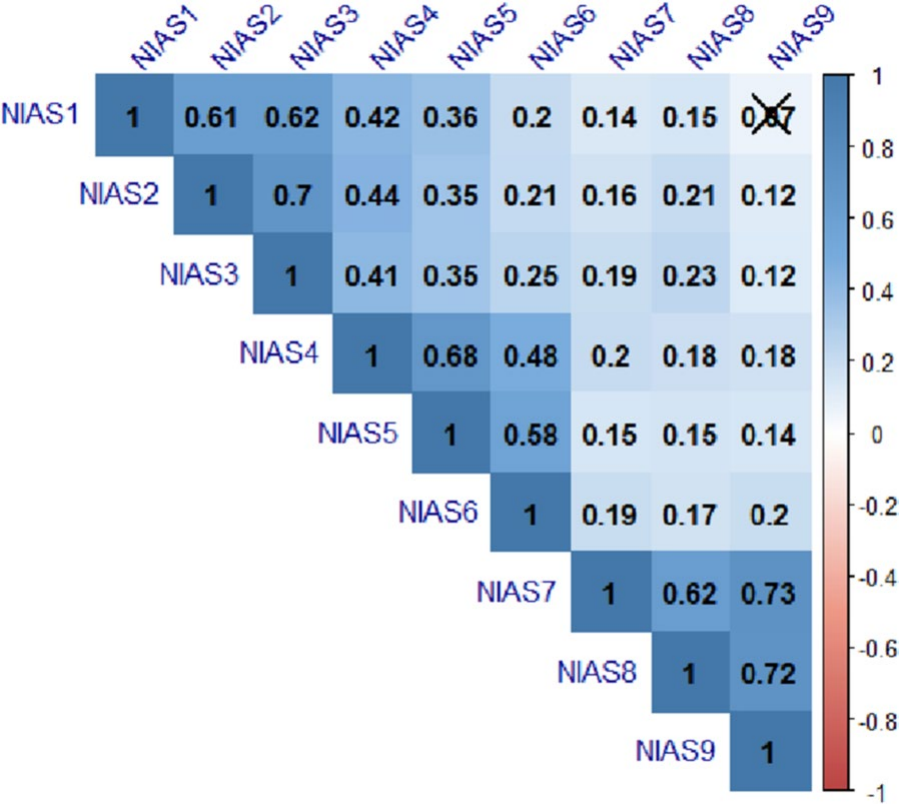
Correlation heatmap of items of the NIAS scale

In Fig. 1, the heat map turns blue if there is a positive relationship between the variables, and the heat map turns red if there is a negative relationship. If there is no significant relationship between the variables, it does not take on any color, and there is a cross (X) over the cell (r = 0.07). Except for the insignificant relationship between the first and ninth items of the NIAS scale, the other items were significantly related to each other. The heat map for the correlation analysis is given in Fig. 1.

#### Test–retest

Correlation analysis was performed to determine the relationship between the two administrations. Statistically significant and strong associations were found for Picky eating (r = 0.94, *p* < 0.001), Appetite (r = 0.94, *p* < 0.001), Fear (r = 0.82, *p* < 0.001), and the total score (r = 0.95, *p* < 0.001). A very high similarity was found between the two applications in terms of the total score and sub-dimensions of the scale.

## Discussion

In this study, the validity of the parent form of the Turkish NIAS scale in children was investigated, and its psychometric properties were examined. The Turkish NIAS scale was shown to be valid and reliable. Through the reliability analysis, the NIAS demonstrated good internal consistency. The three-factor model of the Turkish NIAS was found to be in an acceptable structure by CFA analysis. NIAS scores were shown to be higher in underweight participants. The NIAS-parent version subscales showed expected convergent and divergent validity with the CEBQ, EDEQ-S, and RCADS scales in children.

In the study that the NIAS was developed and first validated, Cronbach's α for the Total score was 0.90 [7], 0.86 in the Chinese NIAS validity study [10], 0.88 in the Polish study [29], 0.84 in the Mexican study [30], and alpha 0.81 in the Turkish NIAS. In addition, the three-factor structure in our study also exhibited internal consistency similar to other studies.

In addition, the three-factor NIAS model was acceptable in the CFA analysis. NIAS-picky eating, NIAS-appetite, and NIAS-fear subscales of the goodness of fit indices were compatible. This model supports the separation of ARFID in the DSM-5 as in the original study [7]. In the scale, NIAS-picky eating indicated the presentation of selective/neophobic ARFID, NIAS-appetite the presentation of lack of interest, and NIAS-fear the presentation of fear of negative consequences [31].

In factor covariances and correlations between NIAS subscales, Picky eating, Appetite, and Fear subscale scores were found to be correlated with each other. These relationships show that the scale harmoniously evaluates the three presentations to be measured. Good inter-item correlation coefficients show that the three subscales measure the ARFID presentation in a standard way [7]. In addition, as in the studies conducted in the USA (adults), China (college undergraduates), and Mexico (adolescents), no gender differences were found in the NIAS total score and subscale scores [10, 30].

In our study, only NIAS-appetite was associated with a low BMI percentile, and underweight children had higher scores on this subscale. Still, no such relationship could be shown between NIAS-picky eating and NIAS-fear. The results for Turkey, located at the intersection of Europe and Asia, were in line with the literature on the relationship between selective eating and body weight in Western countries, as expected [USA (e.g., [7, 32], United Kingdom (e.g., [33]), and Australia (e.g., [34]]. In China, NIAS-appetite and NIAS-picky eating were associated with lower BMI in young adults, which the authors speculated was due to a less obesogenic food environment in China compared with the US, Australia, and Europe [10]. The literature for the USA/Europe/Australia is somewhat mixed, but systematic reviews suggest no relationship between children's picky eating and BMI in these countries [35]. Understandably, being less motivated to eat and restricting volume (i.e., Appetite ARFID symptoms) is protective against obesity in most countries, but restricting variety may only be protective against obesity in certain food environments. In highly obesogenic food environments such as the USA and Mexico, there is sometimes a slight positive correlation between picky eating and BMI [36]. Picky eaters, particularly young children, may indeed prefer certain processed foods with high calories. These foods are often highly palatable due to their high fat, sugar, and salt content, making them more appealing to picky eaters [37].

As hypothesized, there was evidence of divergent validity with the EDEQ-S, a measure of eating disorder symptoms maintained by cognitive restraint, weight and shape concerns, and fear of weight gain [11]. The presence of a small positive correlation between NIAS-picky eating and the EDEQ-S is consistent with findings from the adult NIAS. NIAS validation studies from treatment-seeking samples with eating disorders suggest that while the NIAS is valid and reliable in this population, it has a relatively poor ability to discriminate between ARIFD and other EDs due to the tendency of patients with non-ARFID EDs to endorse ARFID symptoms on the NIAS [31]. As a result, it is recommended to use NIAS and EDE-Q together to evaluate both ARFID and other eating disorders for a more complete picture of the eating behaviors and motivations behind them than is offered by either measure alone [31].

On the other hand, the CEBQ is a scale with different subscales that measures appetitive traits, early-emerging physiological, emotional, and behavioral responses to food and eating that are linked to weight gain (food approach traits) or protection against obesity (food avoidance traits) [15]. The CEBQ measures traits that may represent risk factors for ARFID. As hypothesized, NIAS-appetite showed positive correlations with food avoidance subscales and negative correlations with food approach subscales of the CEBQ. This relationship of NIAS-appetite with Appetite's physiological, motivational, emotional, and behavioral dimensions shows that it is consistent with the ARFID symptoms defined in DSM-5 [1]. NIAS-picky eating was negatively associated with enjoyment of food, while it was positively associated with satiety responsiveness, slowness in eating, emotional undereating, and especially food fussiness [38]. When EAT-26 scores were controlled, the direction and significance of the relationship between NIAS-appetite and CEBQ food approach and food avoidance did not change. Similarly, NIAS-picky eating and FF maintained a positive and strongly associated relationship. As in the adult validation study, the NIAS-fear subscale had weak or null relationships with appetitive traits. Whereas picky eating and appetite disturbances are early-emerging traits often first identified before age 5 in patients who develop selective and Appetite ARFID symptoms, fear ARFID has an acute onset often associated with a conditioning event like choking or vomiting [39, 40]. Temperamental risk factors for this ARFID presentation are likely to overlap with risk for anxiety and affective disorders [3].

The rates of psychiatric disorders accompanying ARFID are high, ranging between 57 and 95% [6, 41]. Anxiety disorders are most common in 36–72% [6, 42], and generalized anxiety disorder is the most common comorbid anxiety disorder in youth with ARFID (although this may reflect the base rate of GAD being higher than that of other anxiety disorders) [41, 43]. Mood disorders accompany ARFID with the second frequency between 17 and 33% [42, 44]. In the relationship with RCADS scores, there was a positive relationship between NIAS-appetite, NIAS-picky eating, NIAS-fear, total anxiety, and depression scores. When non-ARFID eating disorder symptoms were controlled, the relationship between Appetite, Picky eating and Fear subscales, and anxiety and depression measures did not change. Picky eating frequently contributes to the symptoms in clinical samples of those diagnosed with ARFID [39, 40, 45, 46]. Besides, picky eating has been hypothesized to be a transdiagnostic indicator of psychopathology in children, as it is associated with high emotional lability, cognitive rigidity, and concurrent symptoms of anxiety and depression [8, 47, 48]. A recent study showed that picky eating is associated with the symptoms of many concurrent psychopathologies in children, and the basis of this relationship is its association with OCD [49]. The present study also had a stronger relationship between picky eating and OCD symptoms compared to other RCADS subscales. This association may be clinically valuable in improving diagnosis and evaluation in the OCD group, which was generally diagnosed lately. Notably, there was also a stronger association between NIAS-fear and RCADS panic disorder compared to other NIAS/RCADS subscales. Although the association between fear-ARFID and panic disorder is poorly understood, there is evidence of symptom and functional overlap between the diagnoses. In the adult NIAS validation sample, there was a strong correlation between NIAS-fear and a measure of visceral sensitivity analogous to anxiety sensitivity in panic disorder [7].

However, it is noteworthy that the GAD scores of RCADS did not correlate with picky eating and appetite scale scores of NIAS. This lack of correlation may be attributed to the complex and heterogeneous presentation of ARFID, which often includes multiple physical symptoms and comorbid psychiatric [6, 50]. Furthermore, the lack of correlation may also be influenced by the unique eating behaviors and attitudes associated with ARFID. Individuals with ARFID may exhibit selective eating patterns, food avoidance based on sensory characteristics, and limited interest in eating, which may not be fully captured by the GAD scores of the RCADS [3, 51]. Future research should continue to explore the specific relationships between anxiety symptoms, eating behaviors, and comorbidities in individuals with ARFID to better understand the lack of correlation observed in this context. The NIAS subscales were diversely correlated with comorbid psychopathology symptoms, highlighting the importance of comorbidity in children with ARFID. Fink et al.’s study [52] at the gastroenterology clinic found that anxiety and depression scores were higher in cases with more severe ARFID symptoms screened by NIAS. The contribution of this data to clinical practice may be for the use of mirtazapine in treatment. Evidence that mirtazapine, which indicates treating adult anxiety and depression, can be used to treat ARFID is increasing daily [53–55]. Although mirtazapine contributes to weight gain by increasing appetite, it may facilitate the treatment of ARFID by treating anxiety and depression, which are often comorbid with ARFID. It may even support cognitive behavioral therapy (CBT) interventions used in treating ARFID. Similarly, a recent study has shown that selective serotonin reuptake inhibitors (SSRIs) and/or hydroxyzine show promise by reducing anxiety in treating ARFID [56].

To the best of our knowledge, this is the first study to investigate the relationship between ARFID symptoms and anxiety, depression, OCD symptoms, and eating behaviors through the NIAS, in addition to the validity study of the Turkish NIAS scale. The results of the study are significant because the study provides a first step towards providing tools to assist in the assessment of ARFID symptoms in young children aged 6–12 years and shows that the NIAS-parent version is a powerful measurement tool in the evaluation of symptoms in Turkish children. The limitations of the study include cross-sectional design, geographic limitations, and data being obtained from a non-clinical sample. In addition, since the age of the children is not suitable for filling out the scales, the parents' filling in the scales may create a bias. This study obtained the children's height and weight based on parental reporting. Studies have demonstrated that the inaccuracy in parents' reporting of their children's height and weight is generally due to underreporting, although it varies depending on the child's age and the country [57]. Studies need to be conducted to calculate the NIAS cut-off value for ARFID, including clinical samples.

## Conclusion

This study shows that the Turkish version of the NIAS is valid and reliable in evaluating ARFID symptoms in children. The fact that three subscales of NIAS reflect ARFID presentations in correlation with DSM-5 makes NIAS more advantageous than other scales. In the psychometric properties of the Turkish NIAS, appetite, picky eating, and fear subscales were associated with anxiety and depression symptoms and food-avoidant eating behavior. Picky eating also had a relationship with OCD symptoms. It was also found that ARFID symptoms were not associated with eating disorder symptoms.

As the awareness of ARFID increases daily, the demand for assessment tools also increases. Longitudinal studies that may contribute to the diagnosis and treatment of ARFID will contribute to the clinical progress of patients. For this reason, there is a need for scales and validity studies that have been developed and tested and can support clinical decision-making.

## Data Availability

The data that support the findings of this study are available from the corresponding author upon reasonable request.
